# Effects of smoke-free government policy in Qingdao, China: Evidence from the path analysis

**DOI:** 10.1371/journal.pone.0289658

**Published:** 2023-08-03

**Authors:** Nan Jiang, Rui Wang, Haiping Duan, Zhenhua Ma, Lingling Huo, Xiaorong Jia, Xiaocen Jia, Fei Qi, Shanpeng Li

**Affiliations:** 1 Department of Epidemiology and Health Statistics, School of Public Health, Qingdao University, Qingdao, Laoshan District, Shandong Province, China; 2 Qingdao Municipal Center for Disease Control and Prevention, Qingdao, Shibei District, Shandong Province, China; 3 Qingdao Municipal Hospital, Qingdao, Shinan District, Shandong Province, China; 4 Qingdao West Coast New Area Center for Disease Control and Prevention, Qingdao, Huangdao District, Shandong Province, China; Rey Juan Carlos University: Universidad Rey Juan Carlos, SPAIN

## Abstract

Smoke-free government(SFG), as a key tobacco control measure, has been added in Healthy China 2030 blueprint and Qingdao started the establishment of the demonstrative SFG in 2020.This study examined the effects of SFG policy on smoking and smoke-free(SF) environment after establishing the demonstrative SFG. This cross-sectional survey selected participants by simple random sampling from party and government agencies in Qingdao (*N* = 3625) and the participants filled in questionnaires online from November 31 to December 15, 2020. We utilized AMOS to set up models to analyze the direct and indirect effects of SFG policy. The findings showed that knowledge of SFG policy was positively associated with SF environment(*β* = 0.29, *P*<0.001) and negatively associated with smoking(*β* = -0.14,*P*<0.001). Knowledge of SFG policy had indirect effects on SF environment through four channels: independent mediation of discouraging smoking and attitude towards SFG policy, as well as chain mediation of these two factors, and perception of tobacco hazards and discouraging smoking, with indirect effects accounting for 33.5% of the total effect. Knowledge of SFG policy had indirect effects on smoking reduction via SF environment and two chain mediation: SF environment and attitude towards SFG policy, perception of tobacco hazards and intention to quit smoking, with indirect effects accounting for 50.2% of the total effect. The results provided the evidence that SFG policy not only had positive effects on creating SF environment but also on reducing smoking. The efficient policy infiltration to individuals played a vital role in the establishment of SFG. Attitude towards SFG policy, discouraging smoking and SF environment were the potential mediators for SFG policy. Findings in this study added more evidence related to effect mechanism of SFG policy and had a positive influence on promoting the implementation SFG policies for China and other countries.

## Introduction

According to WHO’s report, more than 7 million people die from direct tobacco use and about 1.2 million non-smokers die from exposure to second-hand smoke every year [1]. China is the largest producer and consumer of tobacco in the world bearing huge disease and economic burden and curbing the tobacco epidemic has been a major challenge in the current public health issues. After the adoption of the World Health Organization Framework Convention on Tobacco Control (FCTC) in 2005, China has actively implemented the tobacco control plan. In light of FCTC Guidelines for Implementation on Article 8, all state parties are required to provide protection from exposure to tobacco smoke in indoor workplaces and WHO considers the government facilities as the one of the eight types of public places in comprehensive smoke-free (SF) laws [2, 3]. Healthy China 2030 blueprint proposes that tobacco control is a vital part of Healthy China and all government staff should take the exemplary role in creating SF public places and plans to turn government into smoke-free government (SFG) [4].

Qingdao passed the tobacco control ordinance in 2013, which covered all the indoor public places, workplaces, public transport and some outdoor places and complied with the FCTC. In 2019, Qingdao municipal level government agencies were required to be comprehensively smoke-free. To promote the whole SFG and set a model for implementing the SFG policy, Qingdao started the establishment of the demonstrative SFG in May 2020, which integrated SFG policy with health interventions. It offered effective means to address the deficiencies in enforcement of SFG policy, covering wide ranges of targeted measures such as cessation support service, tobacco control activities and education of SF policy, ordinance, health and so on. The establishment of SFG aimed to make the government office buildings meet the standard of SF environment and provide the guidelines of SFG for other cities. We collected the data of the government staff after the establishment of SFG from November 31 to December 15, 2020 to assess the effects of SFG policy by path analysis in this study.

SF environment, which provides protection from exposure to tobacco smoke, is an essential element in tobacco control and creating SF environment is a crucial goal for SFG policy. Previous studies have demonstrated that SF environment has the advantage of reducing the number of cigarettes smokers smoke and cigarette butts discarded on the ground [5, 6]. A Chinese study has found that benefits of SF environment might earn more supports for SFG policy from the government staff [7]. In this study, SF environment, an environmental factor indicating the smoke or cigarette butts in environment and second-hand smoke (SHS) exposure of government staff in government buildings, can reflect the achievements of SFG construction and we explored the possible mechanism of creating SF environment in path analysis.

Present evidence suggested that SF policy contributed to increasing the willingness to quit smoking and reducing tobacco consumption, smoking and exposure to secondhand smoke [8–10]. However, studies regarding SF policy were mainly based on descriptive analysis and the evidence of effect of SF policy tested by the path analysis was limited. One research focusing on SF workplace policy detected the evidence that effect of policy on secondhand smoke (SHS) exposure was mediated through the smoking prevalence and that on quitting intentions was mediated through smoking harm awareness and smoking consumption [11]. The more effect mechanism and possible pathways are still unknown. Meanwhile, prior studies have commonly measured the SF policy from an objective perspective of policy coverage, yet there remains a lack of exploration regarding individuals’ awareness of smoke-free policies in path analysis. Our study utilized the personal knowledge as a means to assess the SFG policy and this measurement allowed us to ascertain the extent of awareness and knowledge of SFG policy of government staff members, providing insights into the infiltration of policy among government officials after establishing the demonstrative SFG.

There is a need to observe the effects of SFG policy on SF environment and smoking behavior in government buildings and provide more evidence for SFG policy. Our study is one of the first to analyze the SFG policy applying structural equation model (SEM) with Chinese data, aimed at exploring the direct and indirect effects of SFG policy through different channels and catalyzing more effective plans for implementation of such policies. Based on the prior research findings jointly with theoretical models: Knowledge, Attitude, Belief and Practice model (KAP model), Theory of Planned Behavior (TPB) and Social cognitive theory (SCT),we made the following hypotheses:

H_1_: SFG policy has positive effect on SF environment in government buildings.
H_2_: SFG policy has positive effect on smoking behavior of government officials in government buildings.
H_3_: SFG policy is related to perception of tobacco hazards, discouraging smoking and attitude towards SFG policy.
H_4_: SFG policy has indirect effects on SF environment and smoking behavior in government buildings mediated by some potential mediators.

## Materials and methods

### Design and participants

This cross-sectional study was conducted after establishment of SFG by filling out the questionnaires from November 31 to December 15, 2020 via an online survey platform (“SurveyStar,” Changsha Ranxing Science and Technology, Shanghai, China). The contents of the questionnaire were referred to the 2018 China Adult Tobacco Survey Questionnaire [12] and the Guide to the Establishment of Smoke-free Party and Government [13]. The final revised version was determined after excluding irrelevant items and expert argumentation. To ensure the validity and practicability of the questionnaire, 100 questionnaires were distributed for pilot survey after the design. To ensure the authenticity and validity of data, questionnaires were filled anonymously and a number was assigned randomly to each participant to ensure anonymity. Additionally, participants were eligible to submit the questionnaires only once and invalid questionnaires were rejected. All questionnaires were distributed online by investigators from Qingdao Municipal Center for Disease Control and Prevention with assistant of government agencies.

Government employees were the target intervention group in establishment of the demonstrative SFG and the survey selected participants by simple random sampling from employees in party and government agencies involved in demonstrative SFG in Qingdao and 3625 government staff members were recruited. Finally, we got the 3518 valid questionnaires and the effective response rate was 97.05%.

### Measurement

#### Individual characteristics

This part involved age, sex, chronic disease and weekly exercise frequency. Male was coded as 1 and female was coded as 2. Age was comprised of five age groups with ‘Under 25 years old’ coded as 1, ‘25 to 34 years old’ coded as 2, ‘35 to 44 years old’ coded as 3, ‘45 to 54 years old’ coded as 4 and ‘55 years old and above’ coded as 5. Regarding the chronic disease, the participants were asked whether they had been diagnosed with chronic diseases and provided with seven options of specific chronic diseases and two other options: other diseases and no chronic diseases. The option ‘no chronic diseases’ was coded as 0 and other options were coded as 1. Weekly exercise frequency provided four options: ‘Less one time’, ‘One to Two times’, ‘Three to five times’ and ‘Six times and more’ and they were coded from 1 to 4. These variables were included into the path analysis as control variables.

#### Perception of tobacco hazards

Perception of tobacco hazards involved two parts: hazards of smoking and SHS. All participants were asked ‘Do you agree with the following sentences?’ and questions were answered with ‘Yes’, ‘No’ and ‘I don’t know’. Questions about perception of smoking hazards were whether smoking would cause stroke, cardiovascular diseases, respiratory diseases and sexual dysfunction; that about perception of SHS hazards were whether SHS would cause coronary heart disease, lung cancer, sudden infant death syndrome, child pulmonary diseases and premature birth. The right option was coded as 1 and other options were coded as 0. The higher total score indicates the better perception of smoking hazards.

#### Knowledge of SFG policy

This measurement indicated whether the government staff members were aware of the SFG policy and how much they knew it. It included three questions: ‘As far as you know, whether Qingdao has implemented the SFG policy’, ‘As far as you know, whether there is a smoking ban in indoor places of your workplace’ and ‘As far as you know, whether there are any punishments or warnings for violating the smoking ban in your workplaces’. The answer options were ‘Yes’, ‘No’ and ‘I don’t know’. The option ‘Yes’ was coded as 1 and other options were coded as 0. The higher total score represents the better knowledge of SFG policy.

#### Attitude towards SFG policy

All participants were asked the question ‘What’s your attitude towards the SF policy in your workplaces?’ and the response options used the 5-point scale ranging from 0 (the least satisfied) to 4 (the most satisfied). The higher score indicates the satisfaction in higher level towards SFG policy.

#### Discouraging smoking

Discouraging smoking reflected the attitude and reaction towards the smoking behavior in government buildings. All participants were asked the question ‘What would you do if you see someone smoking in the office buildings?’ and the response options were ‘Discourage the smoker’, ‘Avoid the smoker’ and ‘I don’t mind’. Only the participants who chose the option ‘Discourage the smoker’ were classified as having a tendency to discourage smoking and this option was coded as 1 as well as other options were coded as 0.

#### SF environment

SF environment was an environmental factor that can reflect the construction of SF environment after implementation of SFG policy and was measured by the question ‘Have you ever seen any people smoking or smelt smoke or spotted cigarette butts in the indoor places of the office buildings over the past 30 days?’ and ‘Have you ever seen any people smoking or smelt smoke or spotted cigarette butts in the following specific places of your office buildings over the past 30 days?’. Specific places included: area outside the office buildings, toilets, corridors, offices, stairways, lobby on the first floor, elevators, meeting room and other places in office buildings. The response options were ‘Often’, ‘Sometimes’ and ‘Never’. The options were scored from 0(Often) to 2(Never). The higher total score represents the better SF environment.

#### Smoker

According to definition of indicators in 2018 China Adult Tobacco Survey Report, participants who reported smoking every day and smoking occasionally were categorized as smokers. Smoker was measured by the question ‘Do you smoke?’ and the response options were: ‘Yes, every day’, ‘Yes, only occasionally’, ‘I have quit smoking’, ‘Never’.

#### Smoking

Smoking referred to the smoking behavior in several places of government buildings. Participants who were classified as smokers were asked ‘Have you ever smoked in the following places of office buildings over the past 30 days?’ and questions were answered with ‘Yes’ and ‘No’. The specific places involved in the items were: area outside the office buildings, toilets, corridors, offices, stairways, lobby on the first floor, elevators, meeting room and other places in office buildings. The option ‘Yes’ was coded as 1 and ‘No’ was coded as 0. The total score was from 0 to 9 with 0 indicating that the participants had smoked in the least places of government buildings and 9 indicating that the participants had smoked in the most places of government buildings.

#### Intention to quit smoking

Intention to quit smoking involved the past cessation attempts and the current quitting intention. The participants who were classified as smokers were asked two questions: ‘Have you ever quit smoking?’ and ‘Are you going to quit smoking?’. The first question was answered with ‘Never’, ‘Once’ or ‘Twice and more’ and they were scored from 0(Never) to 2(Twice and more). The second question was answered with ‘I don’t want to quit’, ‘I will quit smoking but not within 12 months’, ‘I consider quitting smoking within 12 months’ or ‘I consider quitting smoking within a month’ and they were scored from 0(I don’t want to quit) to 3(I consider quitting smoking within a month). The higher total score represents the stronger quitting intention.

### Statistical analysis

A descriptive analysis was performed using SPSS 24.0 (SPSS Inc., Chicago, IL, USA) and the path analysis for effects of SFG policy was computed by IBM SPSS Amos 26.0 (IBM Corporation, Armonk, NY, USA). *P* values were all two-tailed and significant at the level less than 0.05. Frequencies and percentages were used to describe the categorical variables such as sex, age and smoking status with Chi-square test comparing the differences. Pearson correlation matrix was utilized to analyze the correlations among variables (knowledge of SFG policy, perception of tobacco hazards, discouraging smoking, attitude towards SFG policy and SF environment) preliminarily. Correlations among all variables were significant except that between perception of tobacco hazards and attitude towards SFG policy (P = 0.053). The first SEM was conducted to analyze the possible pathways between knowledge of SFG policy, perception of tobacco hazards, discouraging smoking, attitude towards SFG policy and SF environment. Then, we applied the SEM approach to perform the mediation analysis using two models. The first mediation model included all participants to test the indirect effect of knowledge of SFG policy on SF environment and the second mediation model included only smoking participants to test the direct and indirect effect of knowledge of SFG policy on smoking behavior. We got the assessment of normality and the skewness coefficient for each variable was less than 3 and the and kurtosis coefficient for each variable was less than 8. According to the criterion [14], all variables conformed to normal distribution. Maximum likelihood method (ML) is used for parameter estimation in SEM. In mediation analysis, indirect effects of the knowledge of SFG policy on SF environment and smoking behavior were tested by Bootstrap method (5000 replicates) [15]. Modification Indices (MI) correction line method was employed to optimize the models. The model fit indices included Chi-square/degree of freedom (*χ*^*2*^*/df*), goodness of fit index (GFI), adjusted goodness of fit index (AGFI), comparative fit index (CFI), Tucker-Lewis Index (TLI), root mean square error of approximation (RMSEA). Complying with the critical value for adequate model fit, value of *χ*^*2*^*/df* is less than 5, GFI,AGFI, CFI and TLI is above 0.9, RMSEA is less than 0.06 and SRMR is less than 0.08 [16–18].

### Ethics statement

This study was approved by the Institutional Review Board of Qingdao Municipal Center for Disease Control and Prevention. All methods in this study are in line with the principles of the Institutional Research Committee and the Helsinki declaration. The investigators received standardized training and got the verbal informed consents from the participants before sending the link of questionnaire.

## Results

### Descriptive and correlation analysis

Among the 3518 participants, there were 1964 men (55.8%) and 1554 women (44.2%). 12.6% of participants were smokers and smoking rates among men and women were 22.4% and 0.3% respectively with the statistically significant difference(*χ*^*2*^ = 382.845, *P*<0.001). 88.3% of smoking participants and 91.0% of non-smoking participants had the awareness that Qingdao was implementing the SFG policy (*χ*^*2*^ = 3.362, *P* = 0.067). 77.5% of smoking participants and 77.8% of non-smoking participants would discourage the smokers from smoking in government buildings (*χ*^*2*^ = 0.017, *P* = 0.895). 79.3% of smoking participants and 77.9% of non-smoking participants expressed their satisfaction towards the SFG policy (*χ*^*2*^ = 0.483, *P* = 0.489). 78.9% of smoking participants reported that they hadn’t smoked in indoor places of office buildings in the past 30 days when they were surveyed. The detailed results of the descriptive and difference analyses are presented in Table 1.

**Table 1:** Demographic characteristics and variables related to SFG policy of smokers and non-smokers (*N* = 3518).

Variables	Smokers *n*(%)	Non-smokers *n*(%)	*χ* ^*2*^	*P*
**Sex**			**382.845**	**<0.001**
Male	440(98.9%)	1524(49.6%)		
Female	5(1.1%)	1549(50.4%)		
**Age(years old)**			14.021	0.007
<25	19(4.3%)	147(4.8%)		
25–34	95(21.3%)	780(25.4%)		
35–44	134(30.1%)	873(28.4%)		
45–54	138(31.0%)	1016(33.0%)		
≥55	59(13.3%)	257(8.4%)		
**Chronic diseases**			**18.622**	**<0.001**
Yes	136(30.6%)	658(21.4%)		
No	309(69.4%)	2415(78.6%)		
**Exercise frequency per week**			5.813	0.121
Less one time	60(13.5%)	345(11.2%)		
One to Two times	108(24.3%)	897(29.2%)		
Three to five times	130(29.2%)	893(29.1%)		
Six times and more	147(33.0%)	938(30.5%)		
**Awareness of SFG policy**			3.362	0.067
Yes	393(88.3%)	2797(91.0%)		
No	52(11.7%)	276(9.0%)		
**Discouraging smoking**			0.017	0.895
Yes	345(77.5%)	2391(77.8%)		
No	100(22.5%)	682(22.2%)		
**Satisfied with SFG policy**			0.483	0.489
Yes	353(79.3%)	2393(77.9%)		
No	92(20.7%)	680(22.1%)		
**Smoking in indoor places of government buildings**				
Yes	94(21.1%)			
No	351(78.9%)			

Pearson correlation analysis showed that correlations among all variables were significant except that between perception of tobacco hazards and attitude towards SFG policy (*r* = 0.033, *P* = 0.053). Knowledge of SFG policy was positively associated with SF environment (*r* = 0.434, *P*<0.001), perception of tobacco hazards (*r* = 0.189, *P*<0.001), discouraging smoking (*r* = 0.413, *P*<0.001) and attitude towards SFG policy (*r* = 0.290, *P*<0.001). Discouraging smoking (*r* = 0.370, *P*<0.001) and attitude towards SFG policy (*r* = 0.366, *P*<0.001) had positive associations with SF environment. Perception of tobacco hazards had weakly positive but significant correlation with SF environment (*r* = 0.084, *P*<0.001). The correlation coefficients are shown in Table 2.

**Table 2:** Pearson correlations (*N* = 3518).

Correlation	1	2	3	4	5
1. Knowledge of SFG policy	1.000				
2. Perception of tobacco hazards	0.189***	1.000			
3. Discouraging smoking	0.413***	0.141***	1.000		
4. Attitude towards SFG policy	0.290***	0.033	0.256***	1.000	
5. SF environment	0.434***	0.084***	0.370***	0.366***	1.000

***P<0.001

### Path analysis

On the basis of the correlation analysis, the first SEM tested the effects of SFG policy measured by knowledge of SFG policy on SF environment via different possible pathways. Fit indices of the model all met the standards which revealed an optimal fit (*χ*^*2*^*/df* = 3.784, GFI = 0.997, AGFI = 0.989, CFI = 0.988, TLI = 0.969, RMSEA = 0.028, SRMR = 0.019).

**Fig 1:**
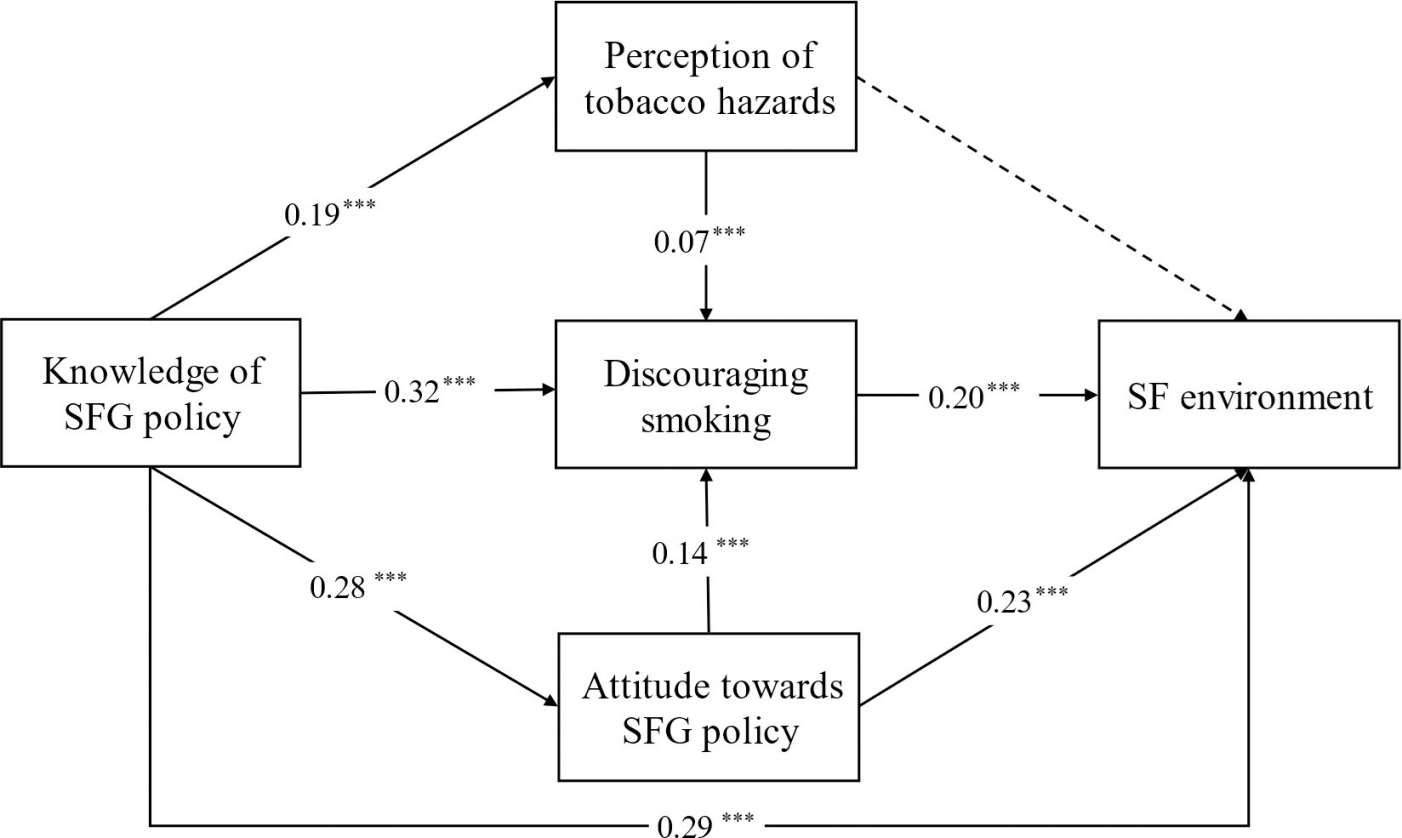
Path model with standardized coefficients. (A) Dotted line indicates the path was not significant at *P* = 0.05, *N* = 3518, ***P< 0.001. (B)Control variables (age, sex, chronic disease and weekly exercise frequency) were included but not reported in the model.

As is shown in Fig 1, better knowledge of SFG policy was associated with better SF environment (*β* = 0.29, *P*<0.001), better perception of tobacco hazards (*β* = 0.19, *P*<0.001), satisfaction in higher level towards SFG policy(*β* = 0.28, *P*<0.001) and discouraging smoking (*β* = 0.32, *P*<0.001). Regarding the SF environment, besides knowledge of SFG policy, discouraging smoking (*β* = 0.20, *P*<0.001) and attitude towards SFG policy (*β* = 0.23, *P*<0.001) were positively and significantly associated with it. Perception of tobacco hazards had no association with SF environment (*P* = 0.435). Attitude towards SFG policy (*β* = 0.14, *P*<0.001) and perception of tobacco hazards (*β* = 0.07, *P*<0.001) were significantly and positively associated with discouraging smoking.

**Fig 2:**
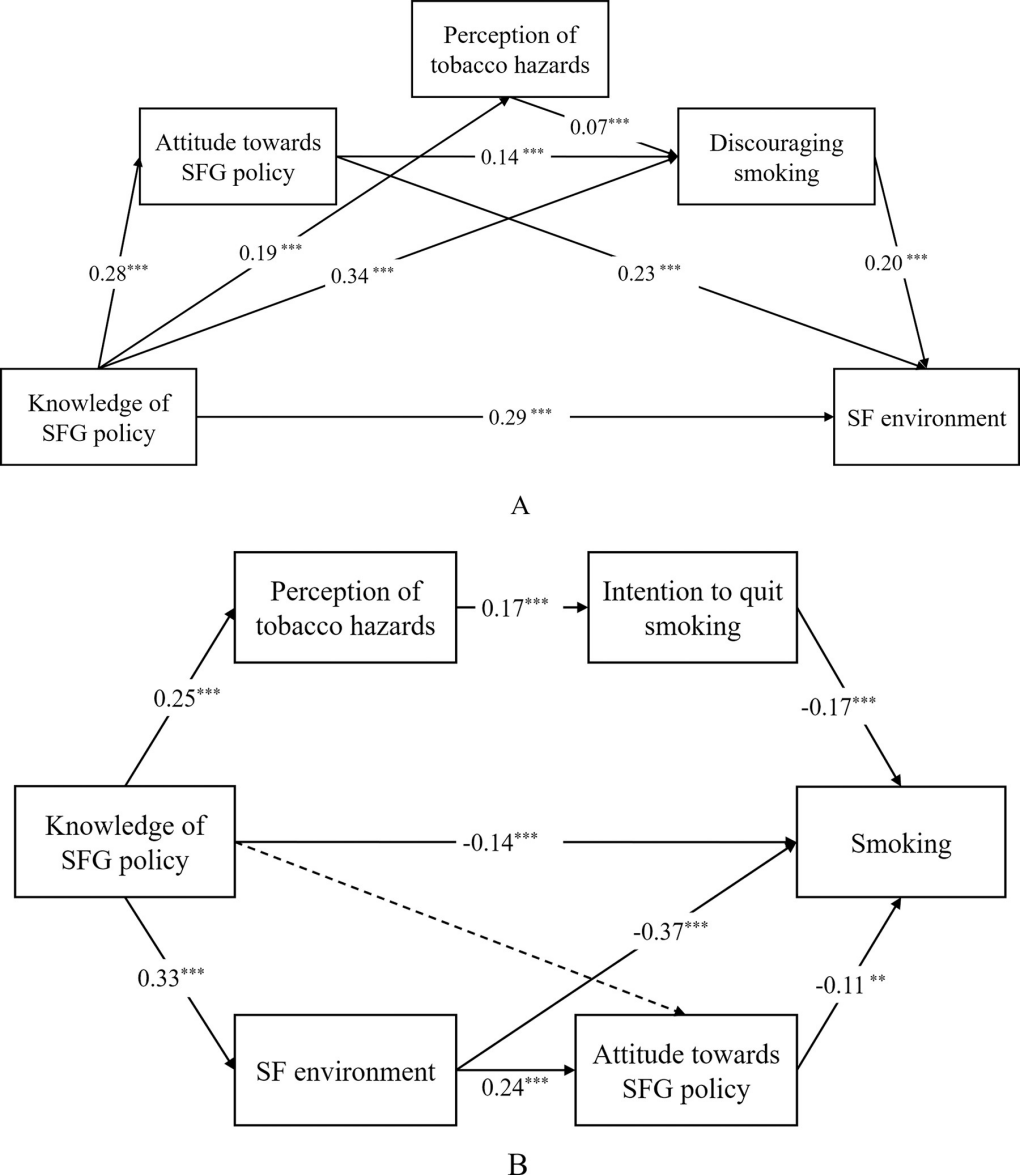
Path models with standardized coefficients. (A) ***P<0.001, **P<0.01. model A: *N* = 3518; model B: *N* = 445. (B) Dotted line indicates the path was not significant at P = 0.05 (C) Control variables (age, sex, chronic disease and weekly exercise frequency) were included but not reported in the models. (D) model A:*χ*^*2*^*/df* = 3.262, GFI = 0.997, AGFI = 0.991, CFI = 0.989, TLI = 0.975, RMSEA = 0.025, SRMR = 0.018; model B: *χ*^*2*^*/df* = 1.638, GFI = 0.979, AGFI = 0.960, CFI = 0.949, TLI = 0.919, RMSEA = 0.038, SRMR = 0.043.

To explore the effects of SFG policy further, we developed two mediation models. As the Fig 2 shows, model A tested the indirect effects of knowledge of SFG policy on SF environment through different pathways. Additionally, model B examined the effects of SFG policy on smoking behavior in government buildings for subgroup of smokers. The results of fit indices showed that these two models all fitted well.

As the Table 3 shows, the mediating effects of each pathway were all significant. In model A, the independent mediating effects of discouraging smoking and attitude towards SFG policy and their chain mediating effect were significant, which indicated that knowledge of SFG policy had indirect effects on SF environment via discouraging smoking and attitude towards SFG policy. Meanwhile, the chain mediating effect of perception of tobacco hazards and discouraging smoking was significant. The mediating effects accounted for 33.5% of the total effect.

**Table 3:** Total, direct, and indirect effects of mediation models.

Model	Effects	Paths	Standardized coefficient(95%CI)
**Model A**	**Direct**	Knowledge of SFG policy →SF environment	0.288***(0.253 ~ 0.328)
	**Indirect**	Knowledge of SFG policy →Attitude towards SFG policy →SF environment	0.065***(0.053 ~ 0.080)
		Knowledge of SFG policy →Discouraging smoking →SF environment	0.071***(0.058 ~ 0.087)
		Knowledge of SFG policy →Attitude towards SFG policy →Discouraging smoking →SF environment	0.008***(0.006 ~ 0.011)
		Knowledge of SFG policy →Perception of tobacco hazards →Discouraging smoking →SF environment	0.003***(0.001,0.004)
	**Total**	Knowledge of SFG policy →SF environment	0.433***(0.400 ~ 0.468)
**Model B**	**Direct**	Knowledge of SFG policy →Smoking	-0.135***(-0.247 ~ -0.037)
	**Indirect**	Knowledge of SFG policy →Perception of tobacco hazards →Intention to quit smoking →Smoking	-0.007***(-0.015 ~ -0.003)
		Knowledge of SFG policy →SF environment →Attitude towards SFG policy →Smoking	-0.009***(-0.015 ~ -0.003)
		Knowledge of SFG policy →SF environment →Smoking	-0.120***(-0.183 ~ -0.074)
	**Total**	Knowledge of SFG policy →Smoking	-0.271***(-0.384~-0.171)

***P<0.001

To examine the effects of SFG policy on smokers, mediation model B was developed for this subgroup. For smokers, knowledge of SFG policy was associated with smoking (*β* = -0.14, *P*<0.001), perception of tobacco hazards(*β* = 0.25, *P*<0.001) and SF environment (*β* = 0.33, *P*<0.001) Perception of tobacco hazards was positively associated with intention to quit smoking (*β* = 0.17, *P*<0.001). Better SF environment was associated with satisfaction in higher level towards SFG policy (*β* = 0.24, *P*<0.001). SF environment (*β* = -0.37, *P*<0.001) and attitude towards SFG policy (*β* = -0.11, *P*<0.001) were negatively associated with smoking. The independent mediating effect of SF environment and chain mediating effects of two paths: SF environment and attitude towards SFG policy, and perception of tobacco hazards and intention to quit smoking were significant, suggesting that knowledge of SFG policy had indirect effects on smoking via these three channels. The mediating effect accounted for 50.2% of the total effect.

## Discusion

The SFG policy has been implemented in Qingdao and the establishment of demonstrative SFG commenced in 2020,contributing to achieving the goal of the whole SFG. This study shed light on effects of SFG policy after the establishment of SFG with Chinese data. Overall, the smoking prevalence among government staff surveyed was 12.6%, far lower than the 33.0% reported in the national survey for government organs in 2008 [19]. This discrepancy suggested that the implementation of tobacco control ordinance in Qingdao since 2013 along with the SFG policy accompanied with health interventions played a significant role in decrease of smoking prevalence. Moreover, a large proportion of smokers reported not smoking in indoor workplaces and most of the participants knew the implementation of SFG policy in Qingdao, indicating that SFG policy was covering the government staff effectively. Nevertheless, there still existed some participants who remain oblivious of the SFG policy in Qingdao. This highlighted the necessity, in research about such SF policies, to not solely consider the objective perspective of policy coverage, but to delve deeper into individuals’ awareness of the policy. This bore significant implications for the establishment of a 100% smoke-free environment.

This study put forward four hypotheses to look into the impact of SFG policy. As the first and second hypotheses predicted, we successfully observed the positive effects of SFG policy on both the creating SF environment and smoking reduction of government officials in government buildings. Our findings are consistent with some previous studies which found that SF policy contributed to lower SHS exposure and lower smoking prevalence [11, 20–24]. Scientific evidences have shown that there is no risk-free level for second-hand smoke and tobacco smoke can drift into adjacent areas no matter the door is open or not [25, 26]. Promoting SF environment and reducing smoking were vital goals in SFG strategy and we did detect the evidence of these beneficial effects.

The further analysis confirmed the third and the fourth hypotheses and found more evidence for effects of SFG policy on SF environment and smoking behavior in government buildings. The evidence showed that better knowledge of SFG policy was associated with better perception of tobacco hazards which indicated that the establishment of SFG could be an effective mean to improve tobacco-related health perception and one previous study exploring the effects of SF policy in workplaces also observed such evidence [11]. Additionally, previous research has shown that knowledge is a central component in health promotion that might affect health behavior change [27] and SFG policy encouraged government officials to stop people smoking in public places. This study found that better knowledge of SFG policy could promote the behavior of discouraging smoking. Evidence from other cities in China showed that the implementation of tobacco control regulation had the positive influence on willingness of discouraging smoking and knowing the relevant provisions in the regulation was a protective factor for the behavior of discouraging smoking [28, 29]. Expanding the coverage of SF policies and targeted education on SF policies and the obligation to discourage smoking in public places will promote the positive behavioral change of people and create a better tobacco control atmosphere in public places. What’s more, this study detected the evidence that the comprehensive knowledge of SFG policy can increase the level of satisfaction towards the SFG policy and some prior research had the similar observation that the knowledge of tobacco control regulations was positively associated with the policy support and SF policy experienced increased support after implementation [30–33]. More effective interventions should be developed to make people understand the rationale for implementing SF policies and encourage the government officials who support the policy to lead by example for people in surroundings and for society, stimulating greater public support for tobacco control policies and increasing social unacceptability of smoking in public places [34–36].

What’s more, we were impressed by some information regarding the effect mechanism of SFG policy. Based on the KAP model and SCT, pathways between SFG policy and SF environment were built and results showed that SFG policy had indirect effect on SF environment through attitude towards SFG policy, perception of tobacco hazards and discouraging smoking. These three factors held significance in the process of translating the policy into SF environment, acting as vital mediators for the SFG policy. The framework of triadic reciprocality where bidirectional relationships occur between behavior, environment and personal factors such as cognitive underpins the SCT [37, 38]. This study found the independent mediating effects of discouraging smoking and attitude towards SFG policy, suggesting that strategic tobacco control plans should be adjusted flexibly to gain the high-level acceptability and support of the SF policy and promote the behavioral change of people to discourage smoking in public places. However, there was a lack of direct effect of tobacco hazards perception on SF environment in first model, indicating that this factor didn’t have independent mediating effect within the mechanism. Fisher suggested that this may be due to the ceiling effect of the information [39], that is, most investigators had a high level awareness of smoking hazards and the direct effect of information has been weakened. Additionally, KAP model posits that the comprehensive knowledge and positive attitudes and beliefs will make people proactively adopt beneficial health behaviors. Attitude towards SFG policy and perception of tobacco hazards were positively associated with discouraging smoking in SEM and their chain mediating effects in the mediation model were significant, demonstrating that SFG policy can effectively establish SF environment by improving satisfaction towards policy, disseminating more knowledge about the tobacco hazards and further promoting the behavior of discouraging smoking. Previous findings showed the relationships between factors that perception of tobacco hazards and knowledge of tobacco control as well as the support for tobacco control regulation had positive effects on dissuading smokers from smoking in public places [28, 40, 41]. The multiple pathways between SF policy and SF environment provided a comprehensive view, elucidating the practical value of these mediators in establishing a SF environment under SF policies. Discouraging smoking, as a crucial mediator for constructing a SF environment, should be prioritized in tobacco control measures. To contribute to the creation of 100% SF environment, it is imperative to carry out in-depth education on dissuasion cognition in public places and strengthen the construction of tobacco control system which provides a safeguard for people to discourage smoking in SF places. Meanwhile, the tobacco control measures should fully consider individuals’ attitude towards SF policy and knowledge of tobacco control to ensure congruence with their knowledge, attitudes, brief and behaviors.

The further analysis was conducted in subgroup of smokers to explore the pathways between knowledge of SFG policy and smoking behavior. The SFG policy had the direct effect on smoking reduction and it also generated the indirect effects by several channels. According to the KAP model and TPB [42], knowledge and health intention are the necessary stages for the change in behavior and we indeed detected this channel that SFG policy reducing the smoking through the chain mediation of perception of tobacco hazards and quitting intention. Some researches detected the similar findings that the tobacco hazards information had indirect effect on smoking behavior and laid groundwork for enhancing willingness to quit smoking and reducing tobacco consumption [43–46]. The repeated replication of research findings attached great importance to the education of tobacco hazards in implementation of SF policy and publicity of tobacco hazards should be strengthened, keeping its practical role in reduction of smoking prevalence.

The evidence of other channels in the subgroup analysis showed something novel that SF environment and attitude towards SFG policy were potential mediators in pathways between knowledge of SFG policy and smoking. SCT highlights the interplay among behavior, environment and personal factors, suggesting that individuals can affect the environment through behavior, while behavior can also be influenced by environmental factors [38]. For smokers, SF environment had positive effect on smoking and it mediated the effect of SFG policy on smoking, suggesting that SFG policy can reduce the smoking behavior in government buildings through creation of SF environment. A study related to smoking of adolescents based on Information-Motivation-Behavioral Skills (IMB) Model revealed that the poor tobacco control environment was associated with more motivation of tobacco use [47]. The influence of different tobacco control environment on smoking behavior varies. SF environment can effectively inhibit smoking behavior and one possible explanation is the normative pressure of conformity that individuals in groups are often reluctant to violate group standards and to be regarded as deviants by other members [48]. SF environment can increase the compliance with smoking bans among smokers and individuals who want to smoke in SF office buildings may consciously refrain from smoking. What’s more, based on KAP model, attitude towards SFG policy was added in the mediation model. Prior finding showed that attitude toward the smoking ban was negatively associated with cumulative cigarette smoking [49] and we also detected similar evidence that more satisfaction towards SFG policy was associated with less smoking behavior in government buildings. Although we failed to find the association between SFG policy and attitude towards policy, we found that SF environment had positive effect on attitude towards SFG policy and SFG policy had indirect effect on smoking via chain mediation of these two factors. Many studies have suggested that public support for SF policy tends to experience an increase following perceiving the benefits brought by SF environment [30, 50, 51]. The chain mediation in this study extended the previous findings, demonstrating that SF environment established under policy could make the SFG policy well received and further bring the better compliance with policy among smokers. Therefore, in order to reduce smoking in SF venues, it should make the best endeavour to promote the establishment of SF environment, encompassing tobacco control measures like prominently posting SF venues signage, rigorously enforcing penalties for smoking infractions and strengthening sustained SF inspections and enforcement efforts. Moreover, augmenting smokers’ acceptance and support towards SF policy will be another effective mean to decrease the smoking prevalence.

In general, SFG policy was perceived to have the positive effect on tobacco control and bring tangible benefits to both smokers and non-smokers in government. The establishment of demonstrative SFG, accompanied with health interventions, made the policy seep into the government staff and contributed to reducing smoking and creating SF environment. The path analysis demonstrated that the individuals’ awareness and knowledge of SFG policy played a crucial role in enhancing the policy effectiveness and unleashing the value of the policy. Efficient infiltration of policy to individuals was conducive to creating SF environment and reducing smoking behaviors. Although SFG policy was implemented within the government system and structures, effects of SFG policy not only existed in government but also in society. Government can leverage its pivotal role and influence to lead by example for society and bring the positive effects generated by policy to the public, catalyzing more public support and compliance with SF regulations. Findings in this study provided a theoretical foundation for optimizing and improving the implementation of SFG policy, offering evidence for promoting the development of SFG in Qingdao and other cities in China. What’s more, an increasing number of countries have adopted the comprehensive SF legislation at national and subnational levels [52]. It needs more evidence to contribute to the continuous promotion of SF policies for many countries but the studies about SFG are limited around the world. Effect mechanism of policy in this study can serve as a reference for implementation of SFG policy in other countries to some extent.

## Limitations

There are still some defects in this study. Firstly, this study utilized the cross-sectional data collected after implementing SFG, whereas the health behavior change is a dynamic process and more longitudinal studies need to be carried out in order to improve tobacco control strategies. Secondly, questionnaire design was referred to some ready-made questionnaire and the information filled was subjective to some extent, which may lead to bias. Therefore, such questionnaires can be optimized in the future, exploring and adding some sensitive indicators and measurement items to reflect the smoking status more comprehensively and objectively.

## Conclusions

Collecting the data after establishment of demonstrative SFG in Qingdao, this study delved into the effects of SFG policy on smoking and SF environment within the context of Healthy China 2030. The results showed that SFG policy not only had positive effects on establishing SF environment but also on reducing smoking. Knowledge of SFG policy was positively associated with perception of tobacco hazards, attitude towards SFG policy and discouraging smoking, suggesting that an in-depth introduction of the SF policy and infiltrating the policy into the personnel were the key in implementation of such SF policies. The further path analysis confirmed that SFG policy contributed to creating SF environment via improving perception of tobacco hazards and attitude towards SFG policy and promoting behavior of discouraging smoking. Additionally, SFG policy had effect on smoking reduction mediated by SF environment and attitude towards SFG policy. The path analysis explicitly showed the mechanism through which SFG policy influenced the environment and smoking behavior, and comprehensively elucidated the relationships among these policy-related and smoking-related factors. Findings provided a theoretical basis for promoting SFG development as well as more effective intervention strategies in Qingdao and other Chinese cities. Moreover, adoption of comprehensive SF legislation has become a trend for global countries and evidence from this study had value in providing reference for SFG policies of other countries.

## References

[1] World Health Organization (2019) WHO launches new report on global tobacco use trends. In: World Health Organization. https://www.who.int/news/item/19-12-2019-who-launches-new-report-on-global-tobacco-use-trends. Accessed 1 Nov 2022

[2] World Health Organization (2009) WHO Report on the Global Tobacco Epidemic, 2009: implementing smoke-free environments. World Health Organization

[3] WHO Framework Convention on Tobacco Control DGO (2017) Guidelines for implementation of Article 8. In: FCTC. https://fctc.who.int/publications/m/item/protection-from-exposure-to-tobacco-smoke. Accessed 1 Nov 2022

[4] (2019) Healthy China Initiative (2019–2030). In: National Health Commission of the People’s Republic of China. http://www.gov.cn/xinwen/2019-07/15/content_5409694.htm. Accessed 23 Apr 2022

[5] (2020) Smoke-free environments: current status and remaining challenges in Australia | PHRP. https://www.phrp.com.au/. 10.17061/phrp3032022 36823799

[6] van Kalmthout D (2023) For a healthy start in life, children need smoke-free environments: Progress of the Generation Smoke-Free campaign in Belgium since its launch in 2018. Tob Prev Cessat 9:06 doi: 10.18332/tpc/155920 36910007 PMC9999196

[7] Lu M, Chen X (2016) A survey on smoking status and related attitudes among government officials in Gusu District, Suzhou City. China Primary Healthcare 30:59–60

[8] Jackson-Morris AM (2019) The contribution of a ‘whole of government’ smoke-free policy on the island of St Helena. Global Health Action 12:1681756 doi: 10.1080/16549716.2019.1681756 31694492 PMC6844416

[9] Hale N, Murphy AM, Adams JR, Williams CM (2017) Effect of a smoke-free policy on staff attitudes and behaviours within an Australian metropolitan health service: a 3 year cross-sectional study. Aust Health Review 41:7 doi: 10.1071/AH15159 27049930

[10] Cho B-Y, Lin H-C, Seo D-C (2020) Effectiveness of Indiana’s Statewide Smoke-Free Indoor Air Law in Reducing Prevalence of Adult Cigarette Smoking. J Prim Prev 41:87–103 doi: 10.1007/s10935-020-00579-z 31953593

[11] Lin H, Liu Z, Chang C (2020) The Effects of Smoke-Free Workplace Policies on Individual Smoking Behaviors in China. Nicotine Tob Res 22:2158–2163 doi: 10.1093/ntr/ntaa112 32597480

[12] Chinese Center for Disease Control and Prevention (2020) 2018 China Adult Tobacco Survey Report. 199

[13] Healthy China Initiative Working Group on Tobacco Control (2020) A Guide to the Establishment of Smoke-free Party and Government. In: Chinese Center for Disease Control and Prevention. https://www.chinacdc.cn/jkzt/sthd_3844/slhd_12890/202007/t20200727_217955.html. Accessed 17 Oct 2022

[14] Kline Rex B (2005) Principles and Practice of Structural Equation Modeling (2nd ed.). New York:THE GUILFORD PRESS, New York London

[15] Preacher KJ, Hayes AF (2008) Asymptotic and resampling strategies for assessing and comparing indirect effects in multiple mediator models. Behav Res Methods 40:879–891 doi: 10.3758/brm.40.3.879 18697684

[16] Malhotra N K, Lopes E, Veiga R T (2014) Structural Equation Modeling with LISREL: An Initial Vision. Brazilian Journal of Marketing

[17] Hu L, Bentler PM (1999) Cutoff criteria for fit indexes in covariance structure analysis: Conventional criteria versus new alternatives. Structural Equation Modeling: A Multidisciplinary Journal 6:1–55

[18] Schumacker R.E, Lomax R.G (2004) A Beginner’s Guide to Structural Equation Modeling (2nd ed.). Mahwah, NJ: Lawrence Erlbaum Associates

[19] Feng G, Jiang Y, Yang Y, Nan Y, Wu X (2011) Survey on Smoking-Related Attitudes and Behavior of Civil Servants across China in 2008. Chin J Prevent Control Chronic Dis 19:230–231+234

[20] Lee JT, Agrawal S, Basu S, Glantz SA, Millett C (2014) Association between smoke-free workplace and second-hand smoke exposure at home in India. Tob Control 23:308–312 doi: 10.1136/tobaccocontrol-2012-050817 23525121 PMC3701026

[21] Azagba S (2015) Effect of smoke-free patio policy of restaurants and bars on exposure to second-hand smoke. Preventive Medicine 76:74–78 doi: 10.1016/j.ypmed.2015.04.012 25913419

[22] Gao J, Zheng P, Gao J, Chapman S, Fu H (2011) Workplace smoking policies and their association with male employees’ smoking behaviours: a cross-sectional survey in one company in China. Tobacco Control 20:131–136 doi: 10.1136/tc.2010.036335 21097936 PMC3045522

[23] Jia X, Wang R, Wu Y, Jia X, Qi F (2022) Evaluation of the intervention effect of the establishment of smoke-free demonstration institutions in Qingdao. Modern Preventive Medicine 49:1813–1817

[24] Tang H, Yang L, Luo X, Ma W (2023) Analysis of the Implementation and Effectiveness of the Smoke-Free Government Initiative in Kunshan City from 2020 to 2022. Jiangsu Journal of health care 25:24–25

[25] Office on Smoking and Health (US) (2006) The Health Consequences of Involuntary Exposure to Tobacco Smoke: A Report of the Surgeon General. Centers for Disease Control and Prevention (US), Atlanta (GA) 20669524

[26] Fu M, Fernández E, Martínez-Sánchez JM, et al (2016) Second-hand smoke exposure in indoor and outdoor areas of cafés and restaurants: Need for extending smoking regulation outdoors? Environ Res 148:421–428 27131796 10.1016/j.envres.2016.04.024

[27] Finney Rutten LJ, Meissner HI, Breen N, Vernon SW, Rimer BK (2005) Factors associated with men’s use of prostate-specific antigen screening: evidence from Health Information National Trends Survey. Prev Med 40:461–468 doi: 10.1016/j.ypmed.2004.07.011 15530599

[28] Yao H, Liu J, Ye X, Yang Y (2014) Willingness to discourage smoking and the influencing factors among staffs in public places in Guangzhou city. Chin J Prev Contr Chron Dis 22:419–422

[29] Liu X, Tang G, Peng L, Wu F, He 中臣, Zhang R, et al. (2022) Willingness to dissuade smoking and its influencing factors of catering workers in the main urban area of Chongqing. Modern Preventive Medicine 49:2623–2628

[30] Fong GT, Hyland A, Borland R, et al (2006) Reductions in tobacco smoke pollution and increases in support for smoke-free public places following the implementation of comprehensive smoke-free workplace legislation in the Republic of Ireland: findings from the ITC Ireland/UK Survey. Tob Control 15 Suppl 3:iii51–58 doi: 10.1136/tc.2005.013649 16754947 PMC2593063

[31] Gilpin EA (2004) Changes in population attitudes about where smoking should not be allowed: California versus the rest of the USA. Tobacco Control 13:38–44 doi: 10.1136/tc.2003.004739 14985593 PMC1747831

[32] Lin B, Lv H, Guo Z (2016) Survey on the Awareness and Attitude of Residents in Shajing Sub-district, Shenzhen, towards the “Shenzhen Special Economic Zone Smoking Control Regulations.” Health Vocational Education Vol. 34:120–122

[33] Chen L, Gao G, Sun T (2022) Survey on effect of tobacco control in public places, Linyi City, 2018 and 2020. Prev Med Trib 28:284–287

[34] Huang W, Zhang N, Luo M, Bai Y, Zeng H, Ma J, et al. (2015) A survey on the cognitive situation of the general public of Chongqing on the policy of smoking control in public. Health Medicine Research and Practice 12:16–20

[35] Thrasher JF, Pérez-Hernández R, Swayampakala K, Arillo-Santillán E, Bottai M (2010) Policy support, norms, and secondhand smoke exposure before and after implementation of a comprehensive smoke-free law in Mexico city. Am J Public Health 100:1789–1798 20466952 10.2105/AJPH.2009.180950 PMC2920995

[36] Thrasher JF, Boado M, Sebrié EM, Bianco E (2009) Smoke-free policies and the social acceptability of smoking in Uruguay and Mexico: findings from the International Tobacco Control Policy Evaluation Project. Nicotine Tob Res 11:591–599 doi: 10.1093/ntr/ntp039 19380383 PMC2688601

[37] Torre D, Durning S (2015) Social cognitive theory: Thinking and learning in social settings. In: Researching Medical Education. John Wiley & Sons, Ltd, pp 105–116

[38] Banduar A (1989) Social cognitive theory. In: Vasta R (ed) Annals of child development: Vol. 6. Six theories of child development. Greenwich: JAI Press, pp 1–60

[39] Fisher WA, Williams SS, Fisher JD, Malloy TE (1999) Understanding AIDS Risk Behavior Among Sexually Active Urban Adolescents: An Empirical Test of the Information-Motivation-Behavioral Skills Model. AIDS and Behavior 03:13

[40] Zhang L, Luo Y, Chen T (2020) Analysis on status of dissuading smoking and influencing factors among medical students. Chinese Journal of Health Education 36:116–120

[41] Zhang H, Guo J, Yang S, Shen Y, Wang R (2015) The Analysis of Passive Smoking and the Status of Dissuading Smoking in Medical College Students After the Tobacco Control Legislation. The Chinese Health Service Management 32:77–79

[42] Ajzen I (1985) From intentions to actions: A theory of planned behavior. In: Action-Control: From Cognition to Behavior. Heidelberg:Springer, Heidelberg, pp 11–39

[43] Jia Z, Xiao L, Zhang Y (2012) Analysis on smoking behaviors influencing factors among middle school students of Taiyuan by structural equation model. Chinese Journal of Health Education 28:722–725

[44] Liu Z, Qin Y, Su J, Luo P, Zhou J, Gong X, et al. (2018) Structural equation model analysis of influencing factors of adolescent smoking prevention behavior in Jiangsu Province based on IMB model. Chin J Health Stati 35:546–548

[45] Wen X, Chen W, Lu C, Zhang C, Luo Y, Deng X, et al. (2006) Analysis on factors influencing the smoking behaviors among male secondary school students under the structural equation model. Chin J Epidemiol 234–237 16792893

[46] Tu M, Nan Y, Wang L, Wang J, Yang Y, Jiang Y (2017) The analysis on awareness rate of tobacco hazard among Chinese adults. Chin J Prev Contr Chron Dis 25:404–408

[47] Zhu C, Cai Y, Ma J, Li N, Zhu J, He Y, Redmon P, Qiao Y (2013) Predictors of Intention to Smoke among Junior High School Students in Shanghai, China: An Empirical Test of the Information-Motivation-Behavioral Skills (IMB) Model. PLoS ONE 8:e80482 doi: 10.1371/journal.pone.0080482 24244690 PMC3828279

[48] Hogg Michael, Vaughan Graham (2010) Essentials of social psychology. NJ: Prentice Hall, London

[49] Haddad C, Sacre H, Hajj A, et al (2020) Comparing cigarette smoking knowledge and attitudes among smokers and non-smokers. Environ Sci Pollut Res Int 27:19352–19362 doi: 10.1007/s11356-020-08162-z 32212070

[50] Li Q, Jiang Y, Zhao G, et al (2009) Support for Smoke Free Policies among Smokers and Non-smokers in Six Cities in China. Chin J Prev Contr Chron Dis 17:8–11+15

[51] Trinidad DR, Gilpin EA, Pierce JP (2005) Compliance and support for smoke-free school policies. Health Educ Res 20:466–475 doi: 10.1093/her/cyg143 15572436

[52] World Health Organization WHO report on the global tobacco epidemic 2019 offer help to quit tobacco use. World Health Organization

